# Eu^3+^ Complex-Protein Co-Crystals as Smart Sensors of Biologically Relevant Cations in Blood

**DOI:** 10.3390/ma19091736

**Published:** 2026-04-24

**Authors:** Miao Qiu, Min Zhang, Runnian Han, Yao Wang, Wei Wang, Yanxin Wang, Jun Li, Christopher D. Snow, Matt J. Kipper, Soo Wohn Lee, Laurence A. Belfiore, Jianguo Tang

**Affiliations:** 1 Institute of Hybrid Materials, National Center of International Research for Hybrid Materials Technology, National Base of International Science & Technology Cooperation, College of Materials Science and Engineering, Qingdao University, Qingdao 266071, China; qm20000515@163.com (M.Q.); zm1998a@163.com (M.Z.); runnianh@163.com (R.H.); wangyaoqdu@126.com (Y.W.); wangwei040901@163.com (W.W.); wangyanxin@qdu.edu.cn (Y.W.); belfiore@engr.colostate.edu (L.A.B.); 2 Guo Yi Materials (Qingdao) Co., Ltd., Qingdao National University Science Park, 127 HuiZhi Qiao Road, Qingdao High Tech Zone, Qingdao 266071, China; 3 School of Materials Science and Engineering, Shanghai University of Engineering Science, Shanghai 201620, China; lijun@sues.edu.cn; 4 Department of Chemical and Biological Engineering, Colorado State University, Fort Collins, CO 80523, USA; matthew.kipper@colostate.edu; 5 Department of Energy and Chemical Engineering, Sun Moon University, Asan 31460, Chungnam, Republic of Korea; swlee@sunmoon.ac.kr

**Keywords:** europium complex, protein, co-crystallization, blood, metal ion sensing

## Abstract

**Highlights:**

- A novel Eu^3+^ complex-doped protein crystal (EC-PC) fluorescent sensor is developed for detecting metal ions in blood with high efficiency.
- Co-crystallization conditions are meticulously optimized, achieving uniform doping of Eu^3+^ complexes within protein crystals and enhancing fluorescence properties.
- The sensor exhibits sensitive and selective quenching of fluorescence by biologically relevant cations, demonstrating high sensitivity to copper ions with dose-dependent response.
- Blood compatibility assays confirm the sensor’s non-hemolytic nature and good anticoagulation properties, ensuring safety for potential biomedical applications.
- This study not only provides a new application perspective for Eu^3+^ complex-doped protein crystals in biosensing but also offers a new strategy for detecting biologically relevant cations in blood, with future potential for clinical sample analysis.

**Abstract:**

This study aimed to develop a novel fluorescent sensor based on Eu^3+^ complex-doped protein crystal (EC-PC) for the efficient detection of metal ions in blood. By meticulously controlling the crystallization and annealing conditions in the co-crystallization strategy, the crystal growth processes were optimized to obtain doped Eu^3+^ complex-co-protein crystalline (EC-PC) structures. Thus, through co-crystallization of hen egg white lysozyme (HEWL) as a model protein and Eu^3+^ complex as fluorescent center, we successfully prepared Eu^3+^ complex-doped-HEWL co-crystals (EC-HC) with excellent fluorescent properties. Further treatment with 4% glutaraldehyde cross-linking enhanced the structural stability of the co-crystals. Moreover, the characteristic of sensitive, selective quenching of EC-PC fluorescence by biologically relevant cations, such as Cu^2+^, Zn^2+^, Mg^2+^, Ca^2+^ and Fe^3+^ ions, set up a smart sensing system in blood. For example, the fluorescence intensity of the crystals at 610 nm, as measured by a UV–visible spectrophotometer, decreases dose-dependently with the concentration of copper ions, thereby validating the sensor’s high sensitivity to copper ion detection. Significantly, we also found that this hybrid protein-based sensor did not induce hemolysis, at various volume concentrations, confirming good anticoagulation in blood. This research not only provides a new perspective on the application of Eu^3+^ complex-doped protein crystals in the field of biosensing but also offers a new strategy for the detection of biologically relevant cations in blood. Future work will focus on further optimizing the sensor’s performance and exploring its potential applications in clinical sample analysis.

## 1. Introduction

The background of co-doped protein crystals and the context of detecting metal ions have provided a solid foundation for our research [1]. In the field of crystal engineering, post-doping (i.e., soaking into pre-existing crystals) and co-crystallization represent two distinct strategies, each with unique advantages and applications. Post-doping is a method that introduces dopants after the crystal growth is complete, allowing for the fine-tuning of specific properties of the crystal [2]. The advantage of this method lies in its flexibility, as dopants can be introduced at any stage of crystal growth, enabling precise control over the crystal characteristics. However, post-doping may not distribute dopants as uniformly throughout the crystal lattice as co-doping [3], which could limit its performance in certain applications. In contrast, co-doping involves the simultaneous introduction of dopants during the crystal growth process, ensuring a uniform distribution of dopants within the crystal, potentially leading to more consistent and predictable optical properties. In our group, co-doping protein crystals with Eu^3+^-complexes and Tb^3+^-complexes has generated doped crystals with value in the field of biosensing, as they can offer higher sensitivity and selectivity, which is crucial for accurately detecting amino acids. When discussing co-doped crystals, we must mention the significant role of lanthanide elements in biosensing. Lanthanide elements, such as europium (Eu^3+^) and terbium (Tb^3+^), have become important components in the field of biosensing due to their unique 4f electronic structures and optical properties [4]. The doping of these elements can significantly impart fluorescence performance to protein crystals, showing great potential in bio-imaging and sensing [5].

Some important biologically relevant cations found in the body, such as copper ion (Cu^2+^), play a multifaceted role in physiological functions within biological systems [6], serving as cofactors in key enzymes, participating in cellular energy metabolism, neurological development, connective tissue formation, and antioxidative defense processes [7]. The role of these ions in cellular metabolism includes being a component of cytochrome c oxidase in cellular respiration and a key player in DNA synthesis and repair through copper-zinc superoxide dismutase. Additionally, copper ions are involved in angiogenesis, cholesterol metabolism, and neural signaling. However, imbalances in copper homeostasis can lead to a range of diseases [8,9,10,11], with Wilson’s disease [12] being a common hereditary condition caused by the accumulation of copper ions in the liver, brain, and other organs, leading to severe health issues. Menkes disease [13] is another condition caused by the disruption of copper metabolism, leading to abnormal distribution of copper ions in the body, affecting brain function and vascular health. Abnormal levels of copper are closely related to the occurrence and progression of these diseases, making the development of accurate and rapid methods for detecting copper ion concentrations of great significance for early disease diagnosis and effective treatment. Current methods for detecting copper ions include atomic absorption spectroscopy, inductively coupled plasma mass spectrometry [14] (ICP-MS), and colorimetry [15], but these methods often suffer from issues such as insufficient sensitivity, complex operation, or high costs.

Lanthanide complexes [16], with their unique optical properties such as long fluorescence lifetimes, large Stökes shifts, and good chemical stability, have become important tools in the field of bio-detection. These properties imbue lanthanide complexes with great potential in bio-imaging [17,18], and sensing [19]. The 4f electrons in lanthanide ions are shielded by outer electrons, resulting in narrow absorption bands and long-lived excited states. This allows time-resolved fluorescence measurements to effectively reduce background fluorescence and enhance detection sensitivity. The large Stokes shift prevents emitted light self-absorption, further improving signal clarity. For various applications these advantages can make lanthanide complexes superior to traditional organic dyes. For instance, Eu^3+^ complexes emit sharp red light, while Tb^3+^ complexes emit green light, enabling high-resolution imaging. In bio-imaging, lanthanide complexes serve as excellent contrast agents. Lanthanide complexes can form sensing systems with proteins and DNA. These complexes can interact with proteins and DNA to create biosensors, which can then be used to detect specific ions or molecules with high selectivity and sensitivity. They can also form aggregates with block copolymers for information storage. In doped crystals, lanthanide ions can interact with metal ions, and this interaction can be detected via fluorescence signal changes, offering a selective and sensitive metal-ion detection method. In summary, lanthanide complexes can play a significant role in bio-detection thanks to their unique optical properties.

In this study, we innovatively combined lanthanide complexes with protein crystals, preparing fluorescent host–guest crystal probes via co-doping. These probe particles leverage the optical properties of lanthanide complexes as well as the biocompatibility and porosity of protein crystals to achieve sensitive and selective detection of blood copper ions. By precisely controlling the doping ratio and crystal growth conditions, we optimized the probe’s performance, ensuring an optimal fluorescence response under physiological conditions. In vitro experiments confirmed the probe’s ability to detect copper ions in simulated blood samples. The probe had a rapid response and high specificity to copper ions, enabling accurate detection even in complex biological samples. Additionally, we assessed the probe’s stability and biocompatibility, confirming its safety and effectiveness for practical applications. In summary, the developed lanthanide complex-doped protein crystal fluorescent probe offers a new strategy for copper ion detection, with potential significance in the early diagnosis and treatment monitoring of related diseases. Future work will focus on further probe optimization and clinical sample validation to promote its application in medical diagnostics.

## 2. Materials and Methods

### 2.1. Materials

The following chemicals were obtained and used without further purification:

Lyophilized hen egg white lysozyme (HEWL) was purchased from Hampton Research, Inc (Aliso Viejo, CA, USA).; 2-acetyltrifluorone (TTA, 98%) was purchased from Aladdin Biochemical Company (Shanghai, China); 1,10-phenanthroline (phen, analytical reagent grade), acetylacetone (ACAC, analytical reagent grade), anhydrous ethanol (CH_3_CH_2_OH, analytical reagent grade), and europium(III) chloride hexahydrate (EuCl_3_·6H_2_O, ≥99.99%) were purchased from the National Pharmaceutical Group Chemical Reagent Company (Shanghai, China).

### 2.2. Preparation and Optimization of Crystallization Conditions

Building upon existing research [20,21], we employed an anti-solvent co-crystallization approach to refine the co-crystallization conditions by modulating the crystallization temperature and duration, as well as the annealing temperature and time. The anti-solvent co-crystallization method is a crystal engineering technique based on solubility modulation. By introducing an anti-solvent (i.e., a solvent in which the target substance is poorly soluble) into the solution of the target substance, the solubility of the solute in the system is reduced, thereby inducing supersaturation and promoting the co-precipitation of the solute and coformer to form co-crystals. During the co-crystallization process, lysozyme from hen egg white (HEWL) was utilized as a model protein, which was combined with a Eu^3+^ complex solution to yield ETP-HEWL crystals. Subsequently, the crystals were treated with 4% glutaraldehyde for cross-linking, thereby enhancing their structural stability [22]. Glutaraldehyde cross-linking is a common practice in crystallography, aimed at solidifying the crystal lattice and reducing the likelihood of degradation or dissolution of the crystals during handling and storage. In the future, alternative cross-linking agents such as glyoxal or 1-ethyl-3-(3-(dimethylamino)propyl)carbodiimide (EDC) could be used to further ensure cytocompatibility [23].

In this study, the concentrations of hen egg white lysozyme and europium chloride solution were both 0.01 M, and the volume ratio for mixing was 1:5.

The rationale behind the selection of HEWL as a model protein is its well-characterized biochemical properties and its frequent use in crystallographic studies [24]. The incorporation of Eu^3+^ into the protein matrix is intended to augment the luminescent characteristics of the protein crystals, offering a foundation for the detection of copper ions via fluorescence quenching. The subsequent cross-linking with glutaraldehyde serves to stabilize the crystal structure—the co-crystallized crystal suspension was transferred into a 4% (*v*/*v*) glutaraldehyde solution, which is crucial for reliable sensing applications.

The optimization of co-crystallization conditions is essential for the production of crystals with desired characteristics for subsequent applications in sensing or structural analysis. The precise control of these parameters is vital for generating crystals suitable for high-resolution structural determination and effective sensing performance [25]. The successful preparation of ETP-HEWL crystals paves the way for their application as fluorescent probes in biological and chemical analysis, where their high sensitivity and selectivity can be exploited.

### 2.3. Blood Compatibility Testing of Crystals

For their application in detecting metal ions in blood, it is crucial to study the interactions of luminescent protein crystals ETP-HEWL with blood. For this experiment, fresh blood samples were collected from healthy rabbits through auricular puncture, and immediately mixed with an anticoagulant, ethylenediaminetetraacetic acid (EDTA), to prevent blood coagulation.

Blood samples were then mixed with ETP-HEWL crystals at various volume concentrations (20%, 30%, 40%, 50%) and incubated at room temperature for a period of 20 min. The volume ratio of the blood sample to the crystal solution was 25:1. The mixture was then centrifuged at −4 °C. Subsequently, the hemolysis rate was measured by comparing the change in optical density (OD) of the blood samples before and after the addition of the crystals at a specific wavelength using a UV–visible spectrophotometer. The hemolysis rate was calculated using Equation (1), where OD stands for optical density. The blank controls were phosphate-buffered saline (PBS) solution and ultrapure water (ddH_2_O).

(1) $$Hemolysis\;rate\;(\%)=\left(\frac{OD_{sample}-OD_{PBS}}{OD_{ddH_{2}O}-OD_{PBS}}\right)\times 100\%$$

This methodological approach ensures a thorough assessment of the ETP-HEWL crystals’ blood compatibility, which is essential for their potential use in biomedical diagnostics and therapeutics [26].

### 2.4. Establishment of Copper Ion Detection Method

Based on the Lanthanide earth complex-doped protein co-crystals, we established a fluorescent sensor for detecting copper ions in blood. We prepared copper ion solutions of different concentrations (1 mg/mL to 10 mg/mL) and then immersed the luminescent crystals in copper ion solutions of different concentrations for the same duration. The crystals were then transferred to glass slides for observation of fluorescence changes under a microplate reader. The sensor utilizes the fluorescence quenching phenomenon when Eu^3+^-doped crystals interact with copper ions, achieving high sensitivity and selectivity in copper ion detection.

## 3. Results and Discussion

### 3.1. Crystal Growth Conditions

**Figure 1:**
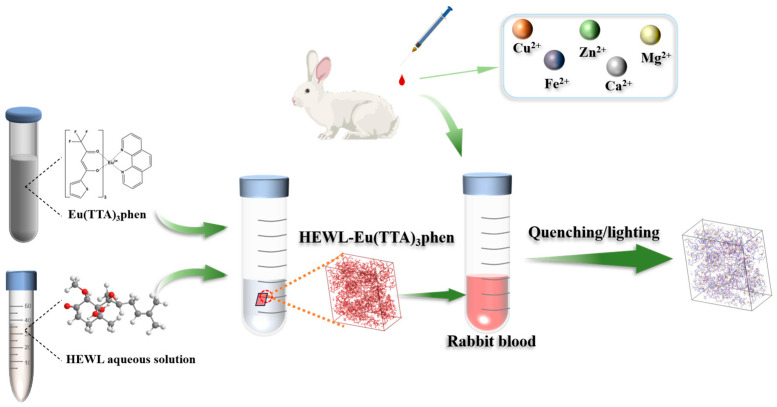
Flow chart for detecting metal ions in blood.

**Figure 2:**
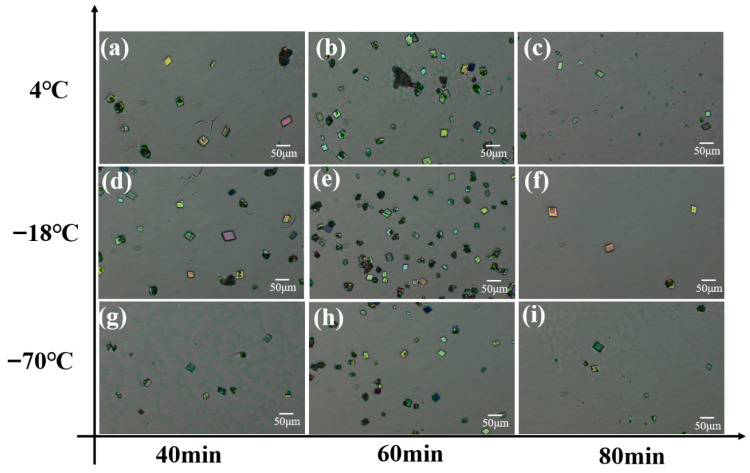
Co-crystal growth versus temperature and time. These images depictspolarized light microscopy images of co-crystals obtained at 4 °C with crystallization times of (**a**–**c**) 40 min, 60 min, and 80 min, respectively. Similarly, the crystallization temperatures for (**d**–**f**) are at −18 °C, and for (**g**–**i**) at −70 °C. The scale bars are 50 μm.

As illustrated in the flow chart (Figure 1), the detection of metal ions in blood using Eu^3+^-complex-doped protein co-crystals involves several key steps. In the process of protein crystallization, controlling the solution conditions is of paramount importance to ensure the acquisition of high-quality crystals. Building on advancements in existing research [27], novel developments in protein crystallography and the field of drug design have shown that the optimization of crystallization conditions often involves the adjustment of various parameters, including protein concentration, temperature, pH value, ionic strength, and the addition of solutes. In our study, we have focused particularly on the impact of crystallization time and temperature on crystal growth and dissolution (Figure 2). All other conditions remain unchanged, including pH, buffer solution, and various reagent concentrations. Our findings indicate that, at a constant crystallization temperature, the longer the duration, the fewer the number of crystals produced. Conversely, when the crystallization time is held constant, among the three tested temperatures, the highest yield of co-crystals was achieved at −18 °C, It is worth mentioning that, due to the low freezing point of ETP, the mixed solution remains in a liquid state during the crystallization process and does not solidify even at −18 °C. This could be due to the fact that lower temperatures can slow down the nucleate on rate, contributing to a more uniform nucleation environment, which may promote the growth of higher quality crystals. Furthermore, at lower temperatures, molecular motion is reduced, which may minimize defects and the incorporation of impurities during the crystal growth process [28]. At the same time, the crystal images captured under a polarizing microscope show that the crystal exhibits refracted light of different colors (Figure 2), confirming that it has different crystal planes.

These perspectives are widely supported in the scientific literature [29]. For instance, studies have explored the influence of temperature on protein crystal growth and have found that lower temperatures can be beneficial for obtaining better crystal quality [30]. Additionally, for certain specific crystal systems, such as covalent organic frameworks (COFs), low-temperature crystallization is also considered an effective method for achieving crystals with high purity and crystallinity [31].

Comprehensive understanding of the factors affecting crystallization provides a foundation for the development of more effective strategies to produce high-quality protein crystals, which are essential for structural biology and drug design applications. The optimization of crystallization conditions is a critical step towards the successful application of protein crystals in various scientific and medical fields.

To demonstrate the potential of crystals as sensors, our objective was to reflect the concentration of metal ions through the fluorescence changes of the luminescent crystals. This approach capitalizes on the unique optical properties of Lanthanide element-doped crystals, which can serve as a sensitive indicator of the presence of metal ions in a biological environment.

To confirm the successful integration of the composite material with the crystals, we initially observed the crystals under ultraviolet light using a polarizing microscope. As shown in Figure 2h, the crystals exhibited superior fluorescence performance, characterized by bright and consistent fluorescence. This observation not only validated the successful doping of Lanthanide elements but also indicated their uniform distribution within the crystal matrix.

**Figure 3:**
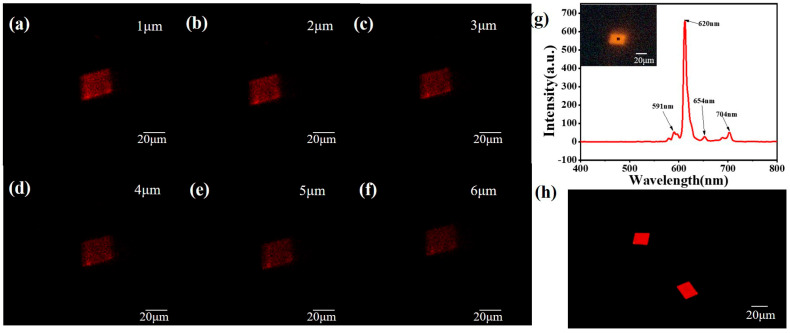
Fluorescence of Doped Co-Crystals. (**a**–**f**) Confocal microscope z-stack is used to evaluate the uniformity of lanthanide element complexes loaded inside crystals. The scale bar is 20 μm. (**g**) CRAIC microspectrophotometry of one such crystal. (**h**) Co-crystals captured under polarizing microscope. The scale bar is 20 μm.

Further confirmation of the effectiveness of the composite material was achieved using a CRAIC microspectrophotometer (CRAIC Technologies, Inc., San Dimas, CA, USA) which allowed us to accurately measure the fluorescence intensity of individual crystals (Figure 3g). The emission spectrum revealed a significant peak around 610 nm, attributable to the Lanthanide ions. The intensity and position of this peak serve as a quantitative measure of the crystal’s response to metal ions, providing a basis for the sensor’s sensitivity.

Additionally, we employed an inverted two-photon confocal microscope to assess the internal fluorescence distribution of the crystals. The interior of the aggregate was observed with 405 nm excitation light for a stack of z planes spaced 1 μm apart. As depicted in Figure 3a–f, a confocal image z-stack showed a fluorescence throughout the crystal interior, consistent with uniform dispersion of the Lanthanide dopants throughout the crystal lattice. This uniformity is crucial for the reliability and reproducibility of the sensor’s response to metal ions.

**Figure 4:**
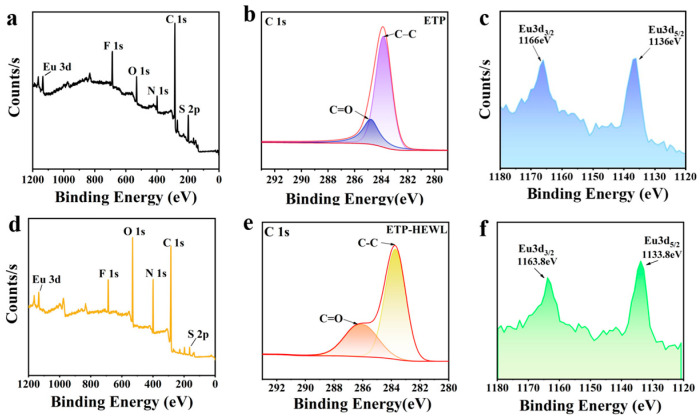
XPS total spectrum (**a**), carbon element peak (**b**), and europium element peak (**c**) of ETP; XPS total spectrum (**d**), carbon element peak (**e**), and europium element peak (**f**) of ETP-HEWL.

As shown in Figure 4c,f, the two main peaks of Eu^3+^ in ETP-HEWL decreased by 2.2 eV compared to ETP, from 1166 eV and 1136 eV to 1163.8 eV and 1133.8 eV. This shift to lower binding energy suggests that Eu^3+^ gained electrons in ETP-HEWL, forming new bonds with HEWL. Figure 4b,e reveal that the C=O binding energy in ETP-HEWL increased by 1.1 eV compared to ETP, rising from 284.9 eV to 286 eV. This increase reflects changes in the local chemical environment of the protein due to Eu^3+^ doping, particularly affecting the electronic structure of the C=O group. This alteration may result from electron transfer or interactions between the f electrons of Eu^3+^ and the p orbitals of oxygen atoms in the protein, modifying the electron density around the C=O group and thus its binding energy.

### 3.2. Annealing of Crystals

In previous studies, the improvement of crystal structure through annealing has been extensively researched. For instance, annealing protein crystals can reduce amorphous regions, enhance crystallinity, and improve mechanical stability [32,33]. For metal–organic frameworks (MOFs) and covalent organic frameworks (COFs), annealing helps control crystal growth rates and morphology, leading to the acquisition of crystals with specific characteristics [34]. Moreover, annealing is commonly used in semiconductors and ceramics to ameliorate the physical and chemical properties of crystals [35].

To further refine the co-crystallized structure, we subjected the crystals to an annealing process. Annealing is a common post-treatment technique in materials science that controls the heating and cooling procedures to eliminate stress within the crystals, reduce defects, and enhance the crystalline quality. In our study, the annealing process was crucial for optimizing the structure of the protein and Lanthanide complex co-crystallized crystals.

Subsequently, we designed a series of annealing experiments as depicted in Figure 4. We systematically varied the annealing temperature and duration to observe the impact of these parameters on the crystal structure. Our experimental results indicated that the annealing treatment can significantly enhance the crystallinity of the crystals, reduce amorphous regions, and thereby improve the quality of the crystals.

**Figure 5:**
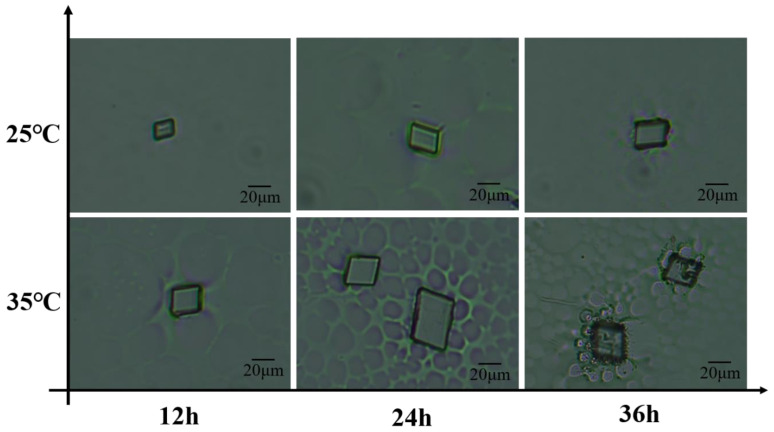
Crystal morphology at different annealing temperatures and times.

We discovered that the optimization of annealing temperature is crucial for reducing defects and impurities within the crystals. By adjusting the annealing temperature, we were able to eliminate stress within the crystals and improve their crystalline quality. In light of these findings, we subjected the crystals to annealing treatments at two different temperatures, 25 °C and 35 °C, with each temperature point having three different annealing durations of 12 h, 24 h and 36 h (Figure 5).

In the comparison experiments conducted at annealing temperatures of 25 °C and 35 °C, crystals annealed at 35 °C exhibited superior morphological characteristics such as crystal size and morphology, and more ordered and intact crystal structures. Additionally, annealing time was identified as a critical factor influencing crystal performance. Experimental results demonstrated that an annealing duration of 24 h is optimal for achieving the best structural optimization and fluorescent properties of the crystals. During this period, the crystals have sufficient time to undergo internal structural adjustments and optimizations, effectively eliminating internal defects, thereby maximizing their crystallinity and optical properties.

Excessively long annealing times, such as 36 h, may lead to changes in the surface properties of the crystals, including edge cracking. These alterations in surface properties could affect the stability and functionality of the crystals in biological environments, thereby limiting their potential applications in biologically relevant fields.

To further confirm the crystalline nature of the samples, we employed transmission electron microscopy (TEM) to scrutinize the internal lattice structure of the crystals. This advanced analytical method provides high-resolution imaging, which is crucial for revealing the precise arrangement of atoms and detecting any crystalline defects. The TEM analysis, in conjunction with the diffraction patterns obtained from X-ray diffraction (XRD), confirmed the single-crystalline structure of the samples.

**Figure 6:**
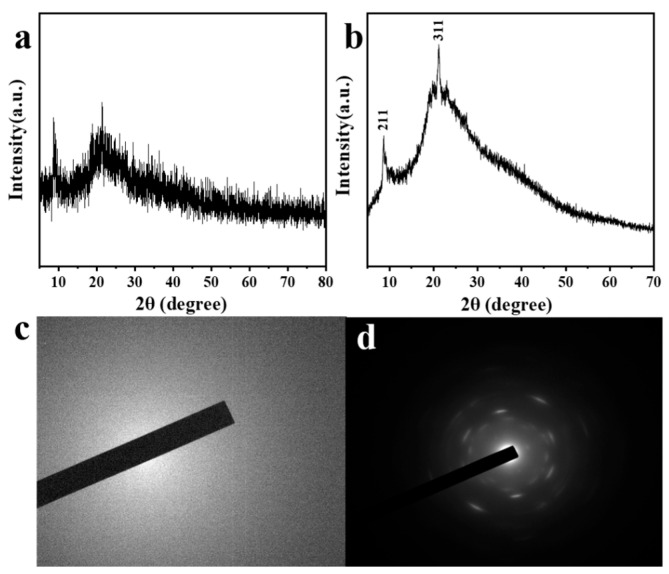
XRD patterns of crystals. Before (**a**) and after (**b**) X min at X °C annealing treatment. X-ray diffraction patterns of crystals before (**c**) and after (**d**) annealing treatment.

The experimental results depicted in Figure 6 offer compelling evidence of the impact of annealing on crystal structure. By comparing the XRD patterns of crystals before (Figure 6a) and after (Figure 6b) annealing, it is evident that the annealed crystals exhibit significantly sharper diffraction peaks. Furthermore, the electron diffraction spectra of the crystals before (Figure 6c) and after (Figure 6d) annealing reveal more pronounced single-crystal diffraction rings in the annealed samples. These observations collectively demonstrate that the annealing process markedly enhances the crystallinity and structural quality of the crystals.

In summary, the annealing treatment at 35 °C for 24 h not only maximizes crystal yield and morphological integrity but also ensures the single-crystalline quality necessary for precise sensing applications. The optimized annealing conditions contribute to the enhanced performance of the fluorescent probes, making them suitable for the detection of metal ions with high sensitivity and selectivity. Future work will focus on exploring the application potential of these crystals in biosensing and their potential in various diagnostic and environmental monitoring scenarios.

Our experimental results are consistent with the existing literature on the impact of annealing on crystal growth. For instance, studies [36] have indicated that during the annealing process, samples tend to form a thermodynamically more stable state, enabling more amorphous components to transition into a crystalline state. Additionally, the influence of annealing time and temperature on crystal growth is supported by other research [37], demonstrating that higher annealing temperatures require shorter durations to achieve full crystallization, while lower temperatures demand longer times. In addition, after annealing, we crosslinked the crystals with glutaraldehyde.

### 3.3. Evaluation of Metal Ion Detection Performance in Blood

**Figure 7:**
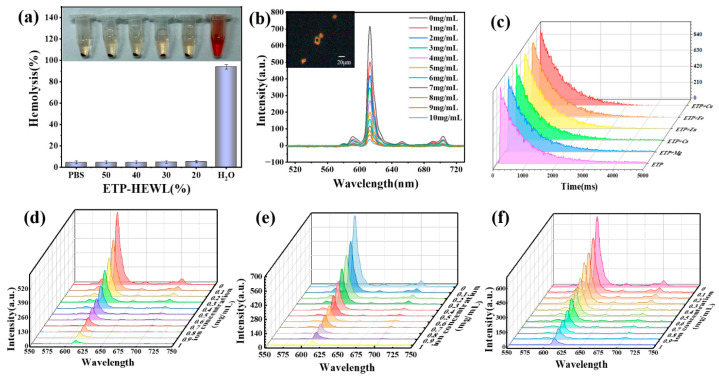
Crystal hemolysis and quenching. No statistically significant effect of varying crystal content (20–50%) on hemolysis (**a**). Quenching of crystals by copper ions of different concentrations with microspectrophotometer collection site marked (black square) in the inset fluorescence microscope image (**b**). Fluorescence lifetime of five metal ions doped with ETP (**c**). Comparison of Quenching Degrees of Copper (**d**), Iron (**e**), and Zinc (**f**).

To test feasibility for the application of ETP-HEWL in blood, we conducted a series of hemolysis assays on ETP-HEWL at various volume concentrations, with water serving as the control. The bar chart (Figure 7a) clearly illustrates the relationship between the volume concentration of the crystals and the hemolysis rate, with water as the control group. The high hemolysis rate of water, at 96%, indicates that water has a destructive effect on red blood cells, leading to the rupture of red blood cell membranes and the release of hemoglobin. With the increase in the volume concentration of the crystals, the hemolysis rate significantly decreased, from 7% at 20% volume concentration to 4% at 60% volume concentration. This trend may be related to the structural characteristics of the crystals, surface charge, or interactions with blood cells. Alternately, the crystals may affect the cells indirectly by affecting the adsorption and activation of blood components [38].

Table S1 presents the normal concentration ranges of some metal ions in human blood as determined by Wishart et al. [39]. Based on these concentration ranges, we conducted fluorescence quenching experiments. Subsequently, we introduced a specific amount of ETP-HEWL (500 μL of 0.01 M crystals) into blood samples (1 mL) and subsequently added copper ion, iron ion, and zinc ion solutions. Utilizing a CRAIC 20/30 PVt microspectrophotometer (CRAIC Technologies, Inc., San Dimas, CA, USA), we monitored a specific spot on the luminescent crystals to assess the quenching effect on the complex’s fluorescence intensity. The experimental data revealed a significant dose-dependent decrease in fluorescence intensity with increasing ion concentration, thereby confirming the sensor’s high sensitivity to copper ions.

Figure 7b shows the effect of different concentrations of copper ions on the crystal fluorescence intensity, as captured under a microcurvature spectrophotometer. The curve clearly demonstrates a distinct concentration-dependent relationship of copper ions on the fluorescence intensity of the crystal. To further elucidate the sensing mechanism, we conducted a series of control experiments to isolate the effects of each ion. The results indicated that copper ions exhibited the most pronounced quenching effect on the fluorescence intensity, followed by iron ions, and then zinc ions (Figure 7d–f). This hierarchy of quenching efficacy may be attributed to the different affinities and interaction dynamics of these ions with the guest europium complexes, the host HEWL crystals, or a combination of both. Furthermore, we compared the effects of five metal ions on the fluorescence lifetime of the crystal at the same concentration. The experimental results are shown in Figure 7c. Copper ions exhibited the most significant impact on the fluorescence lifetime, followed by iron, zinc, calcium, and magnesium ions. This is consistent with the aforementioned quenching results.

To investigate further, we performed kinetic studies to determine the rate constants for the quenching process. The data suggested a static quenching mechanism, where the metal ions form a complex with the fluorophore, thereby reducing its ability to fluoresce. This finding is significant as it provides insights into the interaction between the metal ions and the protein crystals, which is crucial for the design of sensors with specific selectivity.

Additionally, we assessed the selectivity of the ETP-HEWL-based sensor by comparing its response to other biologically relevant ions. The sensor demonstrated minimal cross-reactivity with ions such as calcium and magnesium, further highlighting its potential for specific detection of copper ions in complex biological matrices.

**Figure 8:**
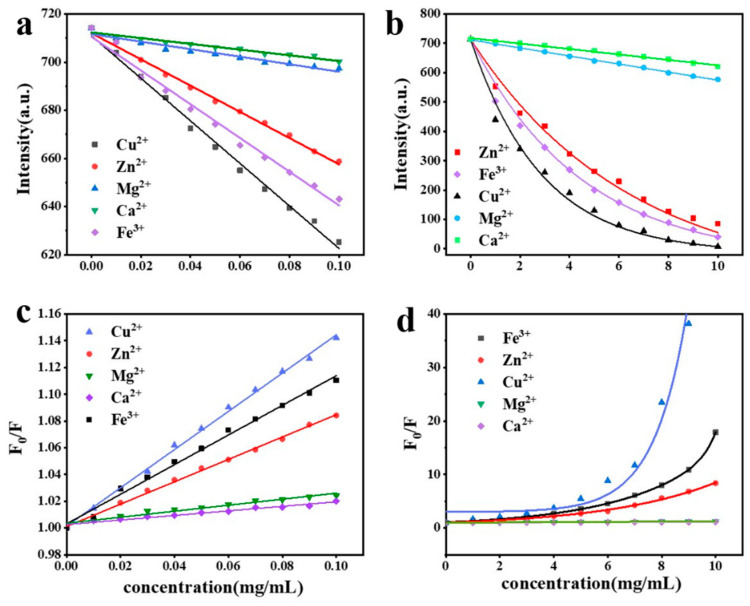
Linear quenching trend within the normal human physiological concentration range (**a**) and the corresponding fitting curve (**c**), as well as the nonlinear quenching trend outside the normal concentration range (**b**) and the corresponding fitting curve (**d**).

When investigating the relationship between fluorescence efficiency (*F*_0_/*F*) and metal ion concentration, we found a complex and subtle connection between the two. To comprehensively analyze this relationship, we introduced the classical Stern Volmer (S-V) equation as an analytical tool, which can accurately describe the quantitative relationship between fluorescence efficiency (*F*_0_/*F*) and metal ion concentration (*C*) (as shown in Figure 8c,d). In the S-V equation system, *F*_0_ represents the initial luminescence intensity of lanthanide complex without interference from metal ions, while F is the actual detection value after the fluorescence intensity of the drug changes in different concentrations of metal ions.

Considering that the normal upper limit of metal ions in human blood is usually around 0.1 mg/mL, we have developed a detailed segmented testing plan based on this concentration as the cut-off point. The use of this segmented testing combined with data fitting helps to accurately depict the complex relationship curve between metal ion concentration and fluorescence efficiency.

Within the normal range of the human body, i.e., within a concentration of 0.1 mg/mL, the equation for fitting different metal ions is

$$\frac{F_{0}}{F}=kC+a$$

In this equation, the parameter *F* reflects the change in fluorescence intensity of the drug within the concentration range of 0 mg/mL to 0.1 mg/mL, and *F*_0_ represents the fluorescence intensity of Eu(TTA)_3_phen. Owing to the influence of different metal ion concentrations, the other coefficients in the equation exhibit distinct characteristics, and their specific values are detailed in Table S3.

From the fitted data (Table S3) and Figure 8c, it can be observed that within the normal physiological concentration range of metal ions in human blood, the five metal ions display distinct characteristic curves with small R values, which sufficiently demonstrates that the protein-Eu^3+^ co-crystalline crystals can be applied to the detection of metal ions in human blood. Through interactions with metal ions, the fluorescence properties of the protein-Eu^3+^ co-crystalline crystals undergo changes, thereby enabling highly sensitive and selective detection of metal ions.

In contrast, an equation for fitting different metal ions outside the normal range of the human body, within a larger concentration range of 0–10 mg/mL, is

$$\frac{F_{0}}{F}=A\times (kC+1)+B_{1}\times exp(\frac{-C}{b_{1}})+B_{2}\times exp(\frac{-C}{b_{2}})+b_{3}$$

In this equation, the parameter *F* reflects the change in fluorescence intensity of the drug within the concentration range of 0 mg/mL to 10 mg/mL. Within this range, the five metal ions exhibit different correlations.

As shown in Figure 8d, the fitted curves for Mg^2+^ and Ca^2+^ nearly overlap, which may be attributed to the negligible quenching effect of both ions. Owing to the influence of different metal ion concentrations, the other coefficients in the equation exhibit distinct characteristics, and their specific values are detailed in Table S4.

From the fitted data (Table S4), it can be observed that beyond the normal physiological concentration range of metal ions in human blood, the five metal ions still display relatively distinct characteristic curves. Specifically, the characteristic curve for Cu^2+^ exhibits a pronounced nonlinear correlation, with a clear inflection in its trend as the concentration varies, suggesting that the interaction mode of copper ions within this concentration range is relatively complex, potentially involving multiple distinct chemical reaction pathways or biological processes. The characteristic curves for Fe^3+^ and Zn^2+^ also deviate from linearity, showing nonlinear correlations. Although the degree of nonlinearity is slightly weaker than that of Cu^2+^, it still indicates that their behavior within this concentration range is not a simple proportional relationship and may be influenced by a combination of factors, such as changes in the coordination environment of the ions or specific binding to other substances.

It is important to note that the exact binding sites of Eu^3+^ in HEWL crystals and whether Cu^2+^ competes with Eu^3+^ for these sites remain to be elucidated. Our XPS data (Figure 4) suggest that Eu^3+^ interacts with carbonyl oxygen atoms of HEWL, as evidenced by the 2.2 eV decrease in Eu^3+^ binding energy and the 1.1 eV increase in C=O binding energy. Regarding Cu^2+^-induced quenching, two mechanisms are plausible: (i) Cu^2+^ competes with Eu^3+^ for the same or adjacent binding sites on the protein, or (ii) Cu^2+^ directly interacts with the Eu^3+^ complex independent of protein mediation. Distinguishing these possibilities requires future structural and biophysical investigations, such as co-crystallization or molecular docking studies.

In this study, we conducted a comprehensive biochemical analysis of rabbit blood to assess its basic physiological state. Given the presence of various components in blood, including proteins, lipids, sugars, and other metal ions, which may interfere with the detection of target metal ions such as Cu^2+^ and Zn^2+^, we performed sample preprocessing in the experiment to eliminate the interference of other ions or substances. Specifically, all samples were adjusted to have the same concentration and volume of blood background [40]. Table S2 presents the biochemical analysis results of rabbit blood, including total protein (TP), albumin (ALB), globulin (GLO), total bilirubin (TBIL), and other indicators. These biomarker test results were within the normal reference ranges, indicating that the rabbit blood samples were not significantly affected by pathological interference.

## 4. Conclusions

In conclusion, the present study successfully developed a highly sensitive and selective fluorescent sensor for the detection of copper ions in blood samples, leveraging the unique properties of Eu^3+^-doped protein co-crystals. In addition to in vitro testing using buffer solutions, the performance of the sensor was evaluated using rabbit blood as a model system, demonstrating its potential for application in clinical sample analysis.

Our findings revealed that the Eu^3+^-doped protein co-crystals were not hemolytic. The anticoagulating nature of the crystals was confirmed through a series of tests. The anticoagulant nature of the crystals was confirmed through a series of tests, ensuring that they do not interfere with the blood’s natural clotting mechanisms. Furthermore, the sensor exhibits high sensitivity toward bioavailable copper ions, with the fluorescence intensity showing a clear dose-dependent decrease as the copper ion concentration increases. This sensitivity was attributed to specific fluorescence quenching interactions between the Eu^3+^-doped crystals and copper ions. The optimization of the sensor performance through controlled annealing at 35 °C for 24 h before crosslinking resulted in crystals with the highest quantity, best morphology, and least amorphous regions. Single crystal X-ray diffraction was done on selected large crystals prepared in this way. We therefore presume that the HEWL packing arrangement is consistent with existing PDB entries. In any event, our crystal growth and annealing optimization work highlights the importance of precise control over crystallization conditions to achieve the desired sensor characteristics. In the experimental study of the interaction between luminescent crystals and five metal ions (copper, iron, zinc, calcium, and magnesium), we observed significant fluorescence quenching. Copper ions, iron ions, and zinc ions had a significant and regular quenching effect on the luminescent crystal, while calcium ions and magnesium ions have almost no quenching effect. Copper ions had the most significant quenching effect on luminescent crystals, followed by iron ions, and then zinc ions, with a potentially useful linear correlation within the normal cation concentration ranges for the human body.

In summary, this study preliminarily validates the fluorescence response characteristics of Eu^3+^-doped protein co-crystals toward Cu^2+^, demonstrating a linear response trend within the physiological concentration range. Future research will systematically evaluate analytical performance parameters such as limit of detection (LOD), limit of quantification (LOQ), repeatability, and recovery, and conduct comparisons with existing detection methods to further elucidate the practical application potential of this sensor. Although the current data support the interaction between Eu^3+^ and the protein and confirm that Cu^2+^ enables detection primarily through fluorescence quenching, the detailed molecular mechanism still requires further elucidation through subsequent structural biology studies.

## Data Availability

The original contributions presented in this study are included in the article/Supplementary Materials. Further inquiries can be directed to the first author.

## Supplementary Materials

The following supporting information can be downloaded at: https://www.mdpi.com/article/10.3390/ma19091736/s1, Table S1: Range of metal ion concentration in human blood; Table S2: Biochemical test results of experimental rabbit blood; Table S3: Fitting data of protein Eu crystal quenching by five metal ions within the normal range of human body; Table S4: Fitting data of protein Eu crystal quenching by five metal ions outside the normal range of human body.
