# Supplementary material for: Eu3+ Complex-Protein Co-Crystals as Smart Sensors of Biologically Relevant Cations in Blood
Source: Materials (Basel). 2026 Apr 24;19(9):1736. doi: 10.3390/ma19091736 (PMC13164826; doi:10.3390/ma19091736)
Supplement: Supplementary file 1 [file materials-19-01736-s001.zip › materials-4235250-supplementary.pdf]

Supporting information

Eu^{3+} Complex-Protein Co-Crystals as Smart Sensors of Biologically Relevant Cations in Blood

Miao Qiu [1], Min Zhang [1], Runnian Han [1,2], Yao Wang [1], Wei Wang [1], Yanxin Wang [1], Jun Li [3], Christopher D. Snow [4,*], Matt J. Kipper [4], Soo Wohn Lee [5], Laurence A. Belfiore [1,4] and Jianguo Tang [1,2,*]

[1] Institute of Hybrid Materials, National Center of International Research for Hybrid Materials Technology, National Base of International Science & Technology Cooperation, College of Materials Science and Engineering, Qingdao University, Qingdao 266071, China; qm20000515@163.com (M.Q.); zm1998a@163.com (M.Z.); runnianh@163.com (R.H.); wangyaoqdu@126.com (Y.W.); wangwei040901@163.com (W.W.); wangyanxin@qdu.edu.cn (Y.W.); belfiore@engr.colostate.edu (L.A.B.)

[2] Guo Yi Materials (Qingdao) Co., Ltd., Qingdao National University Science Park, 127 HuiZhi Qiao Road, Qingdao High Tech Zone, Qingdao 266071, China

[3] School of Materials Science and Engineering, Shanghai University of Engineering Science, Shanghai 201620, China; lijun@sues.edu.cn

[4] Department of Chemical and Biological Engineering, Colorado State University, Fort Collins, CO 80523, USA; matthew.kipper@colostate.edu

[5] Department of Energy and Chemical Engineering, Sun Moon University, Asan 31460, Chungnam, Republic of Korea; swlee@sunmoon.ac.kr

* Correspondence: christopher.snow@colostate.edu (C.D.S.); jtang951@163.com (J.T.)

Table S1 Range of metal ion concentration in human blood

Ionic species	Normal molar concentration in the human body	mass concentration
Ca^{2+}	2.1~2.6 mmol/L	0.08588~0.10608 mg/mL
Mg^{2+}	0.7~1.1 mmol/L	0.017017~0.02631 mg/mL
Cu^{2+}	1.4~1.7 μmol/L	8.895×10^{-5}~1.0795×10^{-4} mg/mL
Zn^{2+}	12~15 μmol/L	0.7846×10^{-3}~0.9807×10^{-3} mg/mL
Fe^{2+}	10~13 μmol/L	0.5585×10^{-3}~1.6755×10^{-3} mg/mL
Fe^{3+}		0.635×10^{-3}~1.905×10^{-3} mg/mL

Table S2 Biochemical test results of experimental rabbit blood

Project Name	Test Results	Reference Unit	Reference Range
AST	26	U/L	42-98
AST/ALT	0.54	—	—
GGT	0.3	U/L	—
ALP	50	U/L	62-209
TBA	3.7	umol/L	0-15
CK	575	U/L	48-554
AMY	276	U/L	200-2500
TG	0.58	mmol/L	0.78-1.76
CHOL	1.7	mmol/L	1.92-4.27
GLU	9.95	mmol/L	4.17-8.06
CRE	98	umol/L	71-159
BUN	7.82	mmol/L	3.6-8.6
BUN/CRE	20	—	—
tCO2	23	mmol/L	20-28
Ca	3.53	mmol/L	1.42-3.77
P	1.27	mmol/L	0.72-3.14
Ca*P	56	—	mg/dL
Mg	0.84	mmol/L	0.38-1.81

TP	63.8	g/L	55-72
ALB	37.4	g/L	25-48
GLO	26.4	g/L	10-47
A/G	1.4		—
TBIL	3.59	umol/L—	0-14
ALT	48	U/L	31-80
AST	26	U/L	42-98
AST	26	U/L	42-98
AST/ALT	0.54	—	—
GGT	0.3	U/L	—
ALP	50	U/L	62-209
TBA	3.7	umol/L	0-15
CK	575	U/L	48-554
AMY	276	U/L	200-2500
TG	0.58	mmol/L	0.78-1.76
CHOL	1.7	mmol/L	1.92-4.27
GLU	9.95	mmol/L	4.17-8.06
CRE	98	umol/L	71-159
BUN	7.82	mmol/L	3.6-8.6
BUN/CRE	20	—	—
tCO2	23	mmol/L	20-28
Ca	3.53	mmol/L	1.42-3.77
P	1.27	mmol/L	0.72-3.14
Ca*P	56	—	mg/dL
Mg	0.84	mmol/L	0.38-1.81

Table S3 Fitting data of protein Eu crystal quenching by five metal ions within the normal range of human body

	K	a	R^2
Cu^{2+}	1.4302	1.0014	0.9974
Fe^{3+}	1.6087	1.0030	0.9921
Zn^{2+}	0.8365	1.0011	0.9970
Mg^{2+}	0.2251	1.0035	0.9509

Ca^{2+}	0.1704	1.0025	0.9557

Table S4 Fitting data of protein Eu crystal quenching by five metal ions outside the normal range of human body

	A	K	B_1	B_2	b_1	b_2	b_3	R^2
Cu^{2+}	0	0	0.2519	0.2289	-1.2079	-1.2079	2.9592	0.9939
Fe^{3+}	0	0	0	0.7385	-0.4278	-3.4264	0.3356	0.9997
Zn^{2+}	0	0	0.4469	0.2818	-4.1279	-4.1279	0.2817	0.9948
Mg^{2+}	0.9913	0.0149	0	0	0	0	0	0.9819
Ca^{2+}	0.9962	0.0239	0	0	0	0	0	0.9966
